# Agaricoglycerides Protect against Hepatic Ischemia/Reperfusion Injury by Attenuating Inflammatory Response, Oxidative Stress, and Expression of NF-*κ*B

**DOI:** 10.1155/2015/142736

**Published:** 2015-04-16

**Authors:** Xiang-qian Zhao, Bin Liang, Yang Liu, Xiao-qiang Huang

**Affiliations:** Hospital & Institute of Hepatobiliary Surgery, Chinese PLA General Hospital, Beijing 100853, China

## Abstract

We have investigated the effects of agaricoglycerides (AG) in a mouse model of hepatic I/R injury. I/R triggered increases/changes in markers of liver injury, hepatic oxidative stress, tumor necrosis factor-*α* (TNF-*α*), interleukin-1*β* (IL-1*β*), and nuclear factor *κ*B (NF-*κ*B). AG significantly reduced the extent of liver inflammation and oxidative stress and also attenuated the NF-*κ*B activation as well as TNF-*α* and IL-1*β* production. Our results indicate that AG may represent a novel protective strategy against I/R-induced injury and inflammatory diseases.

## 1. Introduction

Inflammation has been recognized as a major risk factor for various human diseases. Hepatic ischemia reperfusion (IR) injury often arises from shock and various surgical procedures including liver transplantation. It has been demonstrated that hepatic IR injury can be characterized as an inflammatory response including activation of Kupffer cells, release of proinflammatory cytokines and chemokines, increased expression of adhesion molecules, and leukocyte infiltration [1]. This pathology leads to acute inflammatory responses including activation of Kupffer cells, release of proinflammatory cytokines, and leukocyte infiltration that ultimately causes hepatocellular damage and organ dysfunction [2]. So the pharmacological agents effective for the treatment of inflammatory diseases may also be employed in IR. New anti-inflammatory targets continue to be identified, which is an important area for translational medicine in IR.

Mushrooms have been considered as an edible and medicinal resource for thousands of years.* Grifola frondosa* (GF) is a Basidiomycete fungus belonging to the order Aphyllophorales and family Polyporeceae. It has recently attracted considerable attention for its various physiological activities [3–6]. The extracts from the basidiomas of GF exerted a highly significant hepatoprotective effect by reducing the paracetamol-induced acute elevation of the AST and ALT levels [7]. However, the ingredients accounting for its hepatoprotective effects were not yet understood in terms of modern pharmacological concepts.

**Figure 1:**
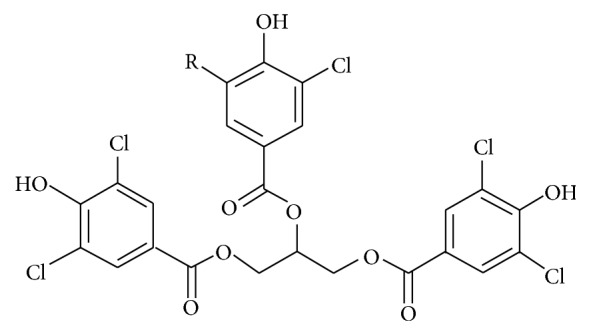
Chemical structures of agaricoglyceride.

The agaricoglycerides (AG) are a new class of fungal secondary metabolites that constitute esters of chlorinated 4-hydroxy benzoic acid and glycerol (Figure 1). Agaricoglycerides showed strong activities against neurolysin, a protease involved in the regulation of dynorphin and neurotensin metabolism, and even exhibited anti-inflammatory and antinociceptive properties [8, 9]. However, the effect of AG on liver I/R injury is poorly understood. In light of the previous protective effects of AG, we therefore investigate whether AG protects the liver from I/R in an experimental mouse model.

## 2. Material and Methods

### 2.1. Agaricoglycerides

The methodology for extraction of AG was described in detail by Han [10]. Briefly, mycelia were separated from fermented mushroom of GF by filtration and extracted twice with acetone in an ultrasonic bath. The extract was filtered, and the acetone was removed* in vacuo* to yield an aqueous residue. This residue was diluted with tap water and subsequently extracted three times with EtOAc. The combined organic phases were dried over Na_2_SO_4_ and evaporated* in vacuo* to yield an oily residue.

### 2.2. Animals

Male C57BL/6J mice (25–30 g) were used in the study. All animal experiments followed the guidelines published by the Ministry of Science and Technology of China. Care was taken to minimize discomfort, distress, and pain to the animals.

### 2.3. Experimental Design

The animals were separated into five groups of ten mice each. The first group served as sham (SHAM). The second group was I/R group (I/R). Group I and group II were treated orally by distilled water for 30 days, respectively. Group III (AG-100), Group IV (AG-300), and Group V (AG-500) were treated orally by AG (100, 300, and 500 mg/kg/day, resp.) for 30 days followed by I/R.

Mice were anesthetized with pentobarbital (65 mg/kg intraperitoneally (ip)). A midline laparotomy incision was performed to expose the liver. The hepatic artery and the portal vein were clamped using microaneurysm clamps. This model results in a segmental (70%) hepatic ischemia as described [11, 12]. The duration of hepatic ischemia was 60 min, after which the vascular clips were removed and liver was reperfused for 2, 6, or 24 h, as indicated. Sham surgeries were identical except that hepatic blood vessels were not clamped with a microserrefine. The liver was kept moist at 37°C with gauze soaked in 0.9% saline. Body temperature was maintained at 37°C using a thermoregulatory heating blanket and by monitoring body temperature with a rectal temperature probe. After reperfusion, blood was collected and liver samples were removed and snap-frozen in liquid nitrogen for determining biochemical parameters or fixed in 4% buffered formalin for histopathological evaluation.

### 2.4. Estimation of Serum ALT and AST

Serum ALT and AST activity was measured colorimetrically using a diagnostic kit (procedure number 505, Sigma Chemical Co., St Louis, MO) according to the instructions provided.

### 2.5. Estimation of Maleic Dialdehyde (MDA)

MDA was determined with thiobarbituric acid (TBA) using the manufacturer's instructions (Nanjing Jiancheng Bioengineering Institute). Total protein content of the samples was analyzed using coomassie blue assay (Nanjing Jiancheng Bioengineering Institute).

### 2.6. Estimation of Liver TNF-*α* and IL-1*β*

Liver samples were disintegrated in 5 volumes of ice-cold RIPA buffer. After incubation on ice for 30 minutes, samples were centrifuged twice at 20,000 ×g for 15 minutes at 4°C. The resulting supernatants were used for assay. The hepatic concentration of TNF-*α* and IL-1*β* was measured by the way introduced by Moon et al. [12] using a commercial ELISA kit (Shanghai Jinma Biological Technology Inc., China) following the manufacture's instruction.

### 2.7. Quantification of NF-*κ*B Activity

Liver tissue extracts were obtained by homogenization of snap-frozen liver tissue in Cell Lysis Buffer, subsequent sonication, and centrifugation. Activated NF-*κ*B was quantified in liver tissue extracts via ELISA-technique using the PathScan Phospho-NF*κ*B p65 (Ser536) Sandwich ELISA Antibody Pair (Shanghai Yubo Biological Technology Inc., China), following the manufacture's instruction. The protein expression levels of NF-*κ*B were measured by western blot analysis.

### 2.8. Measurement of Total Antioxidant Status

The total antioxidant status (TAOS) of liver was determined as previously described by Han [9]. The increase of absorbance at 405 nm was measured by a microplate reader (Shanghai Xunda Medical Technology Inc., China).

### 2.9. Histological Examination of Liver Sections

Liver samples were fixed in 4% buffered formalin (pH 7.2), processed, and embedded in paraffin wax. Sections of 5 mm thickness were then generated and stained with H&E for subsequent light microscope examination. Histological evaluation was performed in a blinded manner. A minimum of two slides per rat were read.

### 2.10. Statistical Analysis

All data were analyzed by a one-way analysis of variance, and the differences between means were established by Duncan's multiple-range test. The data were shown as the mean ± SEM. The significant level of 5% (*P* < 0.05) was used as the minimum acceptable probability for the difference between the means.

## 3. Results and Discussion

In the setting of transplantation, the “injury hypothesis” states that I/R injury to the organ activates a cascade of innate-immunity-dominated proinflammatory responses [13]. The recently described cholinergic anti-inflammatory pathway is a mechanism through which the central nervous system regulates excessive inflammation and limits self-damage [14]. Agaricoglycerides (AG) are a new class of fungal secondary metabolite that has been shown to exert antioxidant and anti-inflammatory effects both in vitro and in various preclinical models of inflammatory disorders [8, 9]. In this study, we demonstrated that AG exerts protective effects against liver I/R reperfusion damage by attenuating major proinflammatory and NF-*κ*B signaling pathways as well as oxidative stress.

### 3.1. Effect of AG on Liver Enzymes

**Figure 2:**
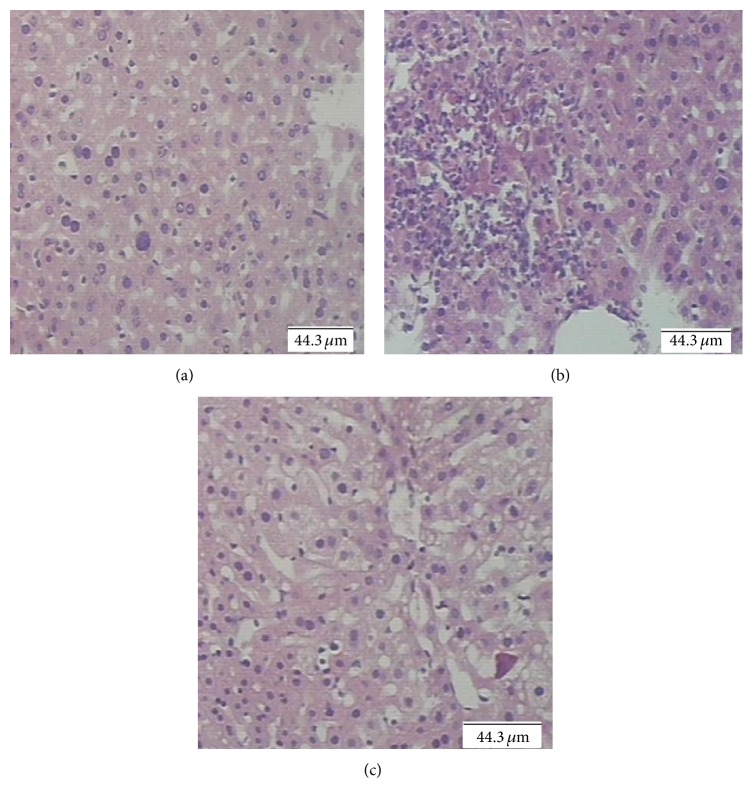
AG decreases I/R-induced neutrophil infiltration after I/R injury. I/R followed by 24 h of reperfusion dramatically increased neutrophil infiltration into the livers (b), which was attenuated by AG pretreatment (c). In livers of sham-operated mice there was no tissue inflammatory cell infiltration (a).

The magnitude of hepatic damage is assessed by measuring the level of released cytosolic transaminases including ALT and AST in circulation, since AST and ALT are sensitive indicators of liver cell injury [15]. In the present study, the I/R-induced increase in serum ALT and AST activity was attenuated by AG.  Table 1 illustrated that a significant increase of ALT and AST level was observed in the I/R group, as compared to the sham mice (*P* < 0.05). AG-500 suppressed this response (*P* < 0.05). On histologic analysis, I/R induced marked congested central veins and blood sinusoids as well as many hepatocytes with deeply stained acidophilic cytoplasm and dark stained nuclei (Figure 2(b)). It was dramatically reduced and became more focal in AG-500 treated mice (Figure 2(c)). The results of the current study provide noticeable evidence that oral administration of 500 mg AG/kg/day for 30 days prior to ischemia protected the liver from I/R injury.

### 3.2. Effect of AG on MDA Level and TAOS

**Figure 3:**
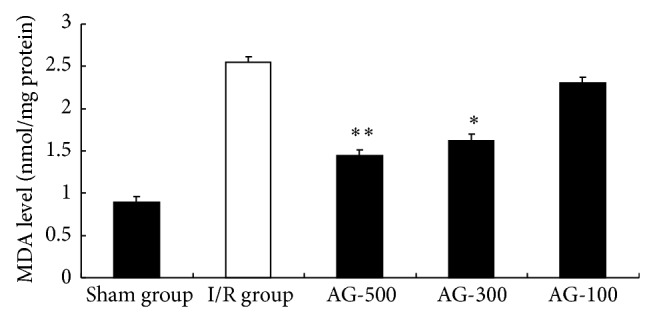
Effect of AG on MDA level. Values represent the mean ± SEM. ^*^ *P* < 0.05 versus I/R group. ^**^ *P* < 0.01 versus I/R group.

Hepatic MDA activity is commonly used as an indicator of liver tissue damage involving a series of chain reactions [16]. Accordingly, we sought to determine whether AG would provide antioxidation by measuring the MDA level. The hepatic tissues from sham animals contained low MDA level. MDA in I/R group was significantly higher than that of sham group (*P* < 0.01). MDA level in AG-300 and AG-500 group was significantly lower than that of I/R group (*P* < 0.05 and *P* < 0.01, resp.) (Figure 3). Growing evidence showed that oxidative stress was involved in liver damage after I/R. MDA is an end-product of free radical formation and lipid peroxidation as well as an index of ROS-mediated injury that results from an imbalance between radical generating and scavenging systems leading to cell membrane impairment or DNA damage [17]. In the present study, administration of AG led to significant reductions in hepatic MDA content compared to levels in the I/R hosts, indicating a reduction in oxidative stress and an enhanced antioxidant state in these hosts' livers. These results indicated that the free radicals being released in the liver were effectively scavenged by AG, which may also account for its anti-inflammatory properties.

The TAOS is an indication of O_2_^−^ and other oxidant species. We measured TAOS activity as an indirect indication of the formation of O_2_^−^ and other oxidant species. The results of hepatic TAOS are shown in Table 2. TAOS in the I/R group was significantly (*P* < 0.01) higher than those in the sham group. Those in the AG-300- and AG-500-treated groups were significantly lower than those in the ethanol-treated group (*P* < 0.05 and *P* < 0.01, resp.). O_2_^−^ is produced by polymorphonuclear leukocytes and macrophages from the enzyme activity of NADPH oxidase and xanthine oxidase at inflammatory sites. The AG groups had the lower level of TAOS activity in comparison to I/R group. We hypothesized that AG produces anti-inflammatory effect through decreasing the levels of TAOS activities.

### 3.3. Effects of AG on Liver TNF-*α* and IL-1*β* Level

**Figure 4:**
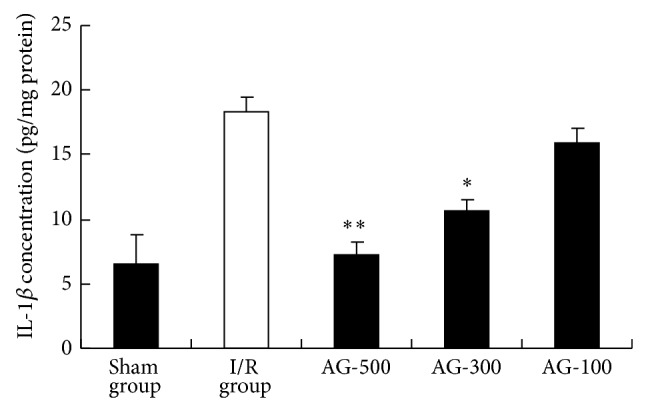
Effect of AG on IL-1*β* level. Values represent the mean ± SEM. ^*^ *P* < 0.05 versus I/R group. ^**^ *P* < 0.01 versus I/R group.

**Figure 5:**
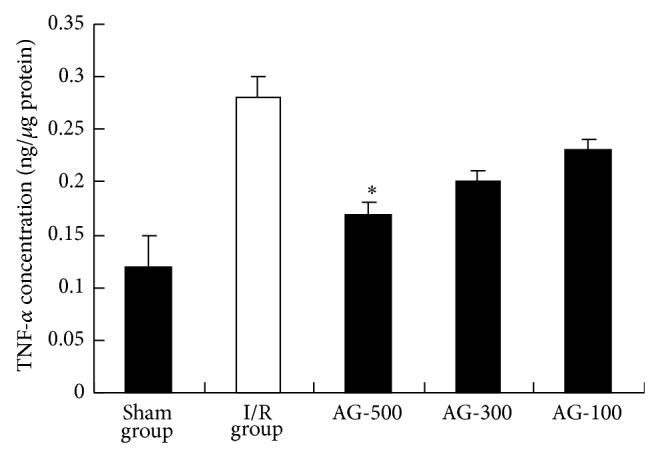
Effect of AG on TNF-*α* level. Values represent the mean ± SEM. ^*^ *P* < 0.05 versus I/R group.

TNF-*α* and IL-1*β* are proinflammatory cytokines which play a critical role in the initiation and progression of hepatic I/R. One of the hallmarks of I/R is the elevated TNF-*α* and IL-1*β* level in serum at the early phase of reperfusion [18]. Therefore, the effect of AG on TNF-*α* production was determined by ELISA. In comparison to I/R group (Figure 4), treatment with AG-300 and AG-500 resulted in a marked decrease in IL-1*β* levels compared with those in I/R group (*P* < 0.05 and *P* < 0.01, resp.). In addition, the levels of TNF-*α* were significantly increased in I/R group (Figure 5). AG-500 suppressed I/R-induced TNF-*α* production (*P* < 0.05). As a proinflammatory cytokine, TNF-*α* can result in liver microcirculation disturbance by interacting with neutrophils and endothelial cells [19]. Besides, IL-1*β* is considered a valid target for therapeutic intervention in diseases associated with tissue remodeling [20]. Thus, downregulating the level of TNF-*α* and/or IL-1*β* will be beneficial for the alleviation of I/R injury.

### 3.4. Effects of AG on Protein Expression of NF-*κ*B

**Figure 6:**
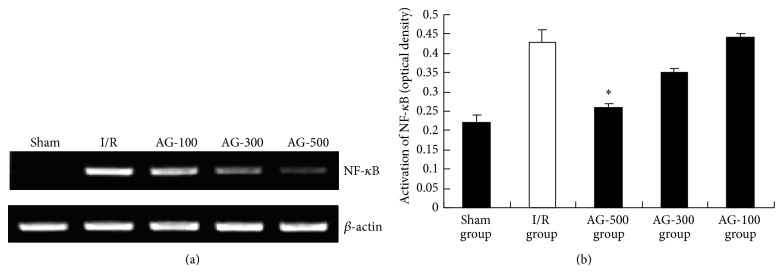
Effect of AG on protein expression of NF-*κ*B. Values represent the mean ± SEM. ^*^ *P* < 0.05 versus I/R group.

To further investigate the mechanisms of AG in hepatic I/R, the protein expression of NF-*κ*B was detected after liver transplantation. Substantially, hepatic I/R induced a predominant increase in nuclear translocation of NF-*κ*B (Figure 6(a)). Conversely, level of NF-*κ*B protein decreased in the nucleus of liver cells of AG-500 group (Figure 6(b)). NF-*κ*B is recognized to have pivotal roles in the inflammatory response to hepatic I/R [21]. Moreover, it is reported that the generation of NF-*κ*B can induce the increase of proinflammatory cytokines, such as TNF-*α* and IL-1*β*, which can further make I/R injury more severe [22].

As expected, nuclear protein levels of NF-*κ*B increased after reperfusion in the present study. The increased NF-*κ*B protein expression was attenuated by AG-500. So we can speculate that AG performs its liver protective effect partly due to the regulation on NF-*κ*B, which can further influence the expression of TNF-*α* and IL-1*β*.

Collectively, our results indicate that AG may represent a novel protective strategy against I/R-induced injury and inflammatory diseases by attenuating the acute and chronic proinflammatory responses, oxidative stress, and expression of NF-*κ*B depending on the dose employed.

## Figures and Tables

**Table 1:** Effect of AG on ALT and AST level.

Different groups	ALT (U/L)	AST (U/L)
Sham group	31.0 ± 3.2^*^	36.0 ± 6.2^*^
I/R group	110.2 ± 21.0	111.5 ± 11.3
AG-100 group	97.1 ± 10.3	92.0 ± 8.0
AG-300 group	83.1 ± 8.8	63.6 ± 9.2
AG-500 group	45.1 ± 8.0^*^	42.6 ± 8.1^*^

Values are shown as means ± SEM, ^*^ *P* < 0.05 versus I/R group.

**Table 2:** Effect of AG on TAOS activity (*µ*M L-ascorbate).

Different groups	TAOS activity (*µ*M L-ascorbate)
Sham group	28.41 ± 3.17^**^
I/R group	80.33 ± 9.32
AG-100 group	72.22 ± 2.78^*^
AG-300 group	64.30 ± 3.38^*^
AG-500 group	56.35 ± 4.33^**^

Values are shown as means ± SEM, ^*^ *P* < 0.05 versus I/R group, ^**^ *P* < 0.01 versus I/R group.
